# Gene signatures predict biochemical recurrence‐free survival in primary prostate cancer patients after radical therapy

**DOI:** 10.1002/cam4.4092

**Published:** 2021-08-28

**Authors:** Qiang Su, Zhenyu Liu, Chi Chen, Han Gao, Yongbei Zhu, Liusu Wang, Meiqing Pan, Jiangang Liu, Xin Yang, Jie Tian

**Affiliations:** ^1^ Beijing Advanced Innovation Center for Big Data‐Based Precision Medicine School of Medicine and Engineering Beihang University Beijing China; ^2^ Clinical Laboratory Medicine Beijing Shijitan Hospital Capital Medical University Beijing China; ^3^ Beijing Key Laboratory of Urinary Cellular Molecular Diagnostics Beijing China; ^4^ Key Laboratory of Big Data‐Based Precision Medicine (Beihang University) Ministry of Industry and Information Technology Beijing China; ^5^ CAS Key Laboratory of Molecular Imaging Beijing Key Laboratory of Molecular Imaging, the State Key Laboratory of Management and Control for Complex Systems Institute of Automation Chinese Academy of Sciences Beijing China; ^6^ CAS Center for Excellence in Brain Science and Intelligence Technology Institute of Automation Chinese Academy of Sciences Beijing China; ^7^ School of Artificial Intelligence University of Chinese Academy of Sciences Beijing China; ^8^ Engineering Research Center of Molecular and Neuro Imaging of Ministry of Education School of Life Science and Technology Xidian University Xi’an, Shaanxi China

**Keywords:** biochemical recurrence‐free survival, gene signature, LASSO‐Cox regression, primary prostate cancer, radical therapy

## Abstract

**Background:**

This study evaluated the predictive value of gene signatures for biochemical recurrence (BCR) in primary prostate cancer (PCa) patients.

**Methods:**

Clinical features and gene expression profiles of PCa patients were attained from Gene Expression Omnibus (GEO) and The Cancer Genome Atlas (TCGA) datasets, which were further classified into a training set (*n* = 419), a validation set (*n* = 403). The least absolute shrinkage and selection operator Cox (LASSO‐Cox) method was used to select discriminative gene signatures in training set for biochemical recurrence‐free survival (BCRFS). Selected gene signatures established a risk score system. Univariate and multivariate analyses of prognostic factors about BCRFS were performed using the Cox proportional hazards regression models. A nomogram based on multivariate analysis was plotted to facilitate clinical application. Kyoto Encyclopedia of Gene and Genomes (KEGG) and Gene Ontology (GO) analyses were then executed for differentially expressed genes (DEGs).

**Results:**

Notably, the risk score could significantly identify BCRFS by time‐dependent receiver operating characteristic (t‐ROC) curves in the training set (3‐year area under the curve (AUC) = 0.820, 5‐year AUC = 0.809) and the validation set (3‐year AUC = 0.723, 5‐year AUC = 0.733).

**Conclusions:**

Clinically, the nomogram model, which incorporates Gleason score and the risk score, could effectively predict BCRFS and potentially be utilized as a useful tool for the screening of BCRFS in PCa.

## Graphical abstract

In this work, we established the risk score consisting of six gene signatures, which can predict biochemical recurrence in primary prostate cancer. Clinically, the nomogram model, which incorporates the Gleason score and the risk score, could effectively predict BCRFS and potentially be utilized as a useful tool for the screening of BCRFS in PCa.

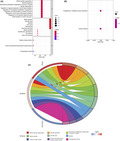

## 1 INTRODUCTION

The second most common male malignancy is prostate cancer (PCa) in the world.^1^ In 2020, estimated new cases and deaths of PCa in the United States will account for 21% and 10%, respectively.^2^ Primary PCa is usually managed with radical prostatectomy (RP) or radical radiotherapy (RT).^3^ Unfortunately, 30%–50% of patients with RT and 20%–40% of patients with RP will develop BCR within ten years.^4^, ^5^ BCR is defined as two consecutive rising prostate‐specific antigen (PSA) values >0.2 ng/ml following RP or >2 ng/ml higher than the PSA nadir value following RT.^6^ It is well known that BCR contributes to distant metastasis. Generally, 24%–34% of men with BCR will progress to metastasis,^7^, ^8^ who should be carefully monitored and endured salvage therapy. Most earlier studies have focused exclusively on the outcomes of PCa following RP or RT. Accordingly, more accurate rapid methods are eagerly needed to identify BCR of primary PCa patients after radical therapy (including RP and RT).

In recent years, notable improvement has been made in precision oncology that applies molecular and medical imaging information to improve the diagnosis and therapy of urological malignancies.^9^, ^10^ In particular, molecular information has outstanding interpretability and discriminative power. For example, gene signatures exhibit an excellent discrimination power for BCR.^11^, ^12^, ^13^ Accumulating evidence has suggested gene deregulation related to the prognosis of PCa, such as CRTC2, MYC, and PTEN.^14^, ^15^, ^16^, ^17^, ^18^ However, gene signatures in the early identification of patients at high‐risk BCR of primary PCa after radical therapy have rarely been reported. Therefore, it is necessary to decipher gene signatures together with underlying molecular mechanisms predicting BCRFS based on genomic information from different platforms.

The current study applied four microarray datasets, which were obtained from GEO and TCGA. Three GEOs were merged as a training set and one dataset from TCGA as a validation set. Afterward, LASSO‐Cox was applied to identify prognostic gene signatures to predict BCRFS and to establish a risk score. Accordingly, the prognostic value of the risk score in both sets was verified. Then, a nomogram was built up to estimate BCRFS time. Finally, GO and KEGG on gene signatures were performed to explore molecular mechanisms and crucial genes.

## 2 MATERIALS AND METHODS

### 2.1 Data preprocessing

In this study, eligible datasets were selected based on the following inclusion criteria: (a) the dataset must include patients with primary prostate cancer (PCa) following radical therapy and (b) patients with clear clinical and pathological information (i.e., gene expression values, Gleason score, BCR event, time to BCR, total follow‐up time). Exclusion criteria were as follows: (a) datasets with a small sample size (*n* < 50) and (b) datasets without complete data for analysis. Gene expression and complete clinical data from 822 (419 samples from GEO and 403 from TCGA) PCa samples that met the inclusion and exclusion criteria were downloaded from the three GEO (GSE70768, GSE70769, GSE116918) and TCGA datasets, serving as the training and validation sets, respectively. The main characteristics of the datasets are shown in Table 1. To ensure data integrity for each indicator, incomplete raw information (i.e., age in the training set) was excluded for further COX analysis. In addition to Gleason score and follow‐up BCR information, the training set included preoperative PSA, clinical T (cT) stage, and radical therapy (RP = 196, RT = 223); meanwhile, the validation set included radical therapy (RP = 403). The characteristics of patients with prostate cancer in the training set and validation set are shown in Table 2. We have made subgroup analyses for every variable in the training set, and the results are shown in Figure S1. Three GEO datasets were merged and applied function “Normalize Between Array” from the R package “limma” for standardization.

**TABLE 1:** Characteristics of the included datasets

Dataset	Country	Number of samples	GPL	Number of genes
GSE70768	United Kingdom	110T	GPL10558	48,107
GSE70769	United Kingdom	86T	GPL10558	48,107
GSE116918	United Kingdom	223T	GPL 25318	121,563
TCGA	N/A	403T	N/A	5,6754

Abbreviations: GPL, Gene Expression Omnibus Platform; GSE, Gene Expression Omnibus Series; N/A, not applicable; T, tumor samples; TCGA, The Cancer Genome Atlas.

**TABLE 2:** The characteristics of patients with prostate cancer in the training set and validation set

Characteristics	Training set (*n* = 419)	Validation set (*n* = 403)
cT stage *n* (%)		
T1	151 (36.0)	150 (37.2)
T2	147 (35.1)	140 (34.7)
T3	117 (27.9)	45 (11.2)
T4	4 (1)	1 (0.3)
Unknow	0 (0)	67 (16.6)
Gleason *n* (%)		
5	2 (0.5)	0 (0)
6	72 (17.2)	37 (9.2)
7	227 (54.2)	198 (49.1)
8	60 (14.3)	56 (13.9)
9	57 (13.6)	109 (27.0)
10	1 (0.2)	3 (0.8)
Biochemical recurrence *n* (%)		
Yes	93 (22.2)	52 (12.9)
No	326 (77.8)	351 (87.1)
Follow‐up time (months, mean ± SD)	45.61±19.49	28.53±17.70

Abbreviations: cT, clinical tumor; SD, standard deviation.

### 2.2 Identification of gene signatures

The batch influence was adjusted for GEO and TCGA by R package “sva.” Gene expression profiling was merged with clinical information for analyses. To select gene signatures with predictive value, LASSO‐Cox regression was applied using the R package “glmnet.”^19^, ^20^ The risk score was founded by weighting individual normalized expression value of gene signature and LASSO coefficient.

### 2.3 Validation of gene signatures

According to the median value of risk score in the training set, both training and external validation sets were classified into high‐risk and low‐risk groups. Kaplan–Meier (K–M) survival curves were drawn by R packages “survival” and “survminer.” Then, logarithmic rank (log‐rank) tests were performed to compare differences in BCRFS time between the high‐ and low‐risk groups. To visualize BCRFS differentially, a heatmap was constructed using the R package “pheatmap.” Multivariate and univariate Cox regression models were established using the R package “survival.” R package “timeROC” was applied to build a t‐ROC curve, which was used to assess the predictive accuracy of the risk score system for BCRFS. Afterward, based on the results of multivariate models, a nomogram was depicted using the R package “rms.” To assess the performance of the nomogram, calibration plots and C‐index were used in both training and validation sets.

### 2.4 Bioinformatical analysis

To estimate the potential functions of DEGs in low‐risk versus (vs.) high‐risk groups, the KEGG pathway and GO annotation were performed using the R package “clusterProfiler.”^21^ Briefly, GO and KEGG annotation sets were derived from the R package “org. Hs.eg.db.” GO reveals the catalogs of biological process (BP), cellular component (CC), and molecular function (MF). All visualizations were produced using R packages “ggplot2” and “GOplot.” After multiple‐test correction, KEGG pathways and GO terms with corrected *P* (*P*.adjust) value <0.05 were considered to be prominently enriched in DEGs.

### 2.5 Statistical analysis

Data analysis was implemented using the R program (version 3.6.3, https://www.r‐project.org) with the following libraries: base‐package, survival‐package, glmnet‐package, survminer‐package, timeROC‐package, limma‐package, rms‐package, and clusterProfiler‐package. Support Vector Machine (SVM) and Random Forest(RF) models were carried out in Python, using the Scikit‐learn (version 0.24.0). BCRFS curves were depicted by Kaplan–Meier plots, and the difference in BCRFS was assessed by the log‐rank test. Multivariate and univariate Cox regression models were used to ascertain independent prognostic factors. Time‐dependent ROC curves were constructed, and AUCs were used to predict the performance of BCRFS in 3, and 5 years, respectively. Nomogram was validated with C‐index. DEGs were defined as differential expression for |logFC| > 0.5 and an adjusted *P* value <0.05. All *P* values <0.05 were considered statistically significant.

## 3 RESULTS

### 3.1 Prognostic gene signatures identification in the training set

**FIGURE 1:**
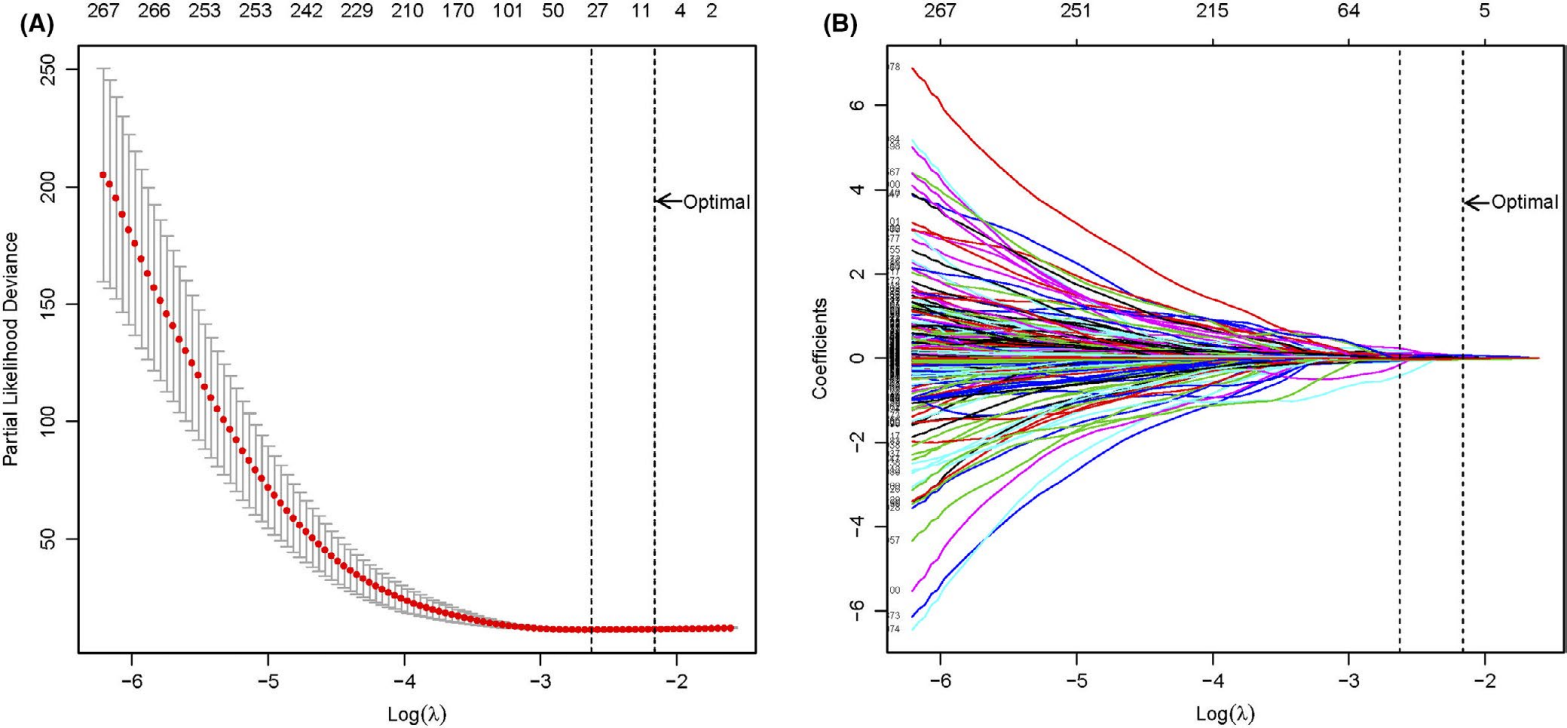
Selection strategy for gene signatures. (A) “Leave‐one‐out” cross‐validation for parameter selection in LASSO‐COX regression models, and the optimal *λ* value of 0.11517381 with log(*λ*) = −2.1613129 was selected; (B) six BCRFS‐associated gene signatures were selected by LASSO‐COX models. LASSO, least absolute shrinkage and selection operator method; BCRFS, biochemical recurrence‐free survival

Gene expression variables in the training set were submitted to high‐throughput LASSO‐Cox proportional hazards regression analysis. All gene variables were reduced to the most useful potential predictors for BCRFS. The optimal *λ* value was chosen by “Leave‐one‐out” cross‐validation, and the *λ* value of 0.11517381 with log(*λ*) = −2.1613129 was selected (Figure 1A). Six BCRFS‐associated gene signatures (NOX4, F12, TPX2, PHYHD1, AURKA, and YIPF1) were identified by LASSO‐COX models. (Figure 1B).

### 3.2 Construction of the risk score

**FIGURE 2:**
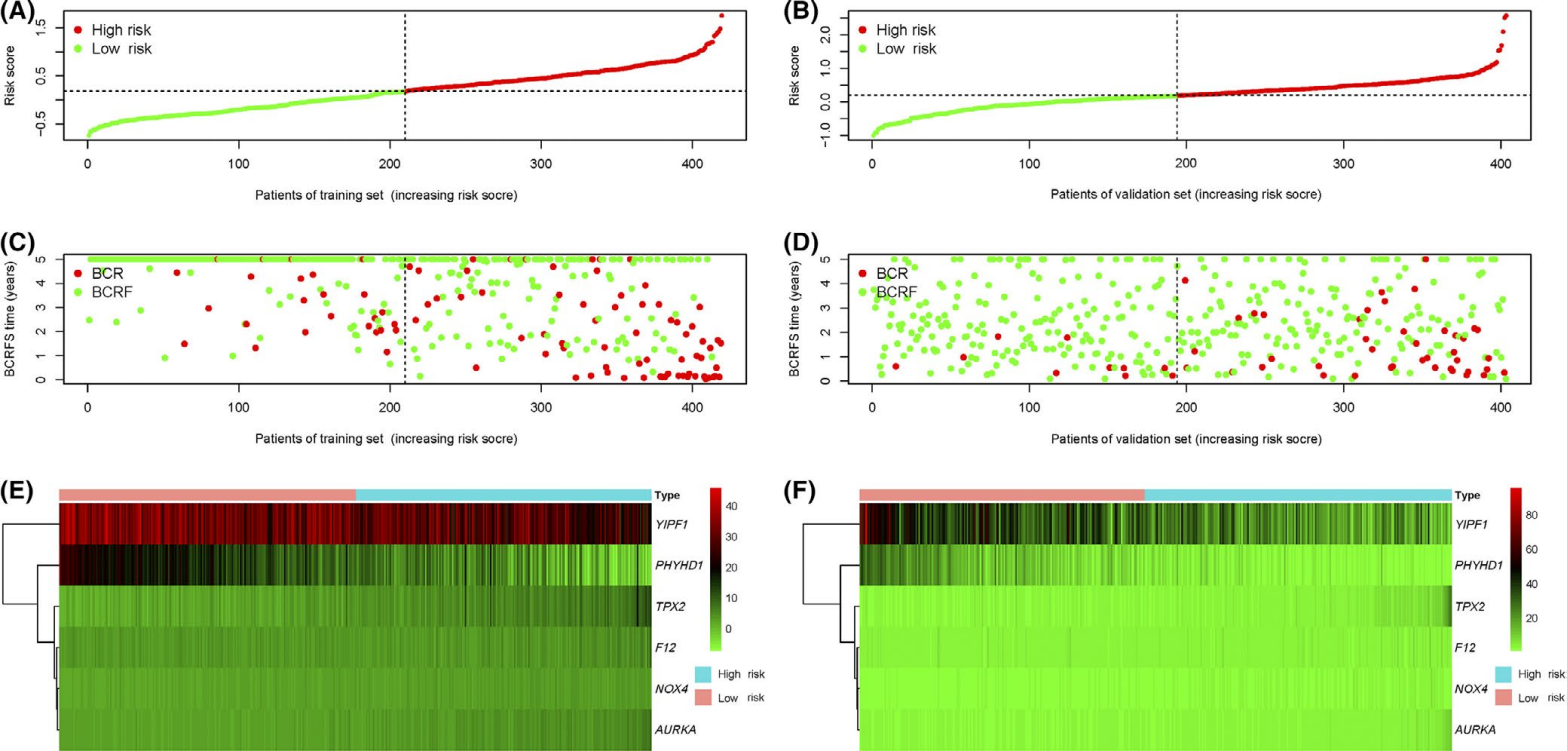
Risk score distribution, BCRFS status, and expression pattern of BCRFS‐associated gene signatures in both cohorts. (A) The scattergram of the risk score in the training set; (B) the scattergram of the risk score in the validation set; (C) BCRFS time/BCR status in the training set; (D) BCRFS time/BCR status in the validation set; (E) the expression pattern of six BCRFS‐associated gene signatures in the training set; (F) the expression pattern of six BCRFS‐associated gene signatures in the validation set. BCRFS, biochemical recurrence‐free survival

The risk score was established by the summating of every gene signature expression value multiplied by its corresponding coefficient, as follows: risk score = (0.046043 × NOX4) + (0.043807 × F12) + (0.066203 × TPX2) + (−0.027543 × PHYHD1) + (0.068834 × AURKA) + (−0.01182 × YIPF1). The expression value of every gene was log2‐transformed and standardized. The distributions of risk scores for the training set and validation set were shown, respectively (Figure 2A,B). The distributions of BCRFS and BCR status for both sets are shown in Figure 2C,D. The risk score was ranked and the high‐risk score indicates poor BCRFS. The median risk score of the training set was used to classify all patients into high‐risk (>0.183427) versus low‐risk (<0.183427) groups. The heatmaps of six prognostic genes expression values were presented in Figure 2E,F.

### 3.3 Validation of the risk score

**FIGURE 3:**
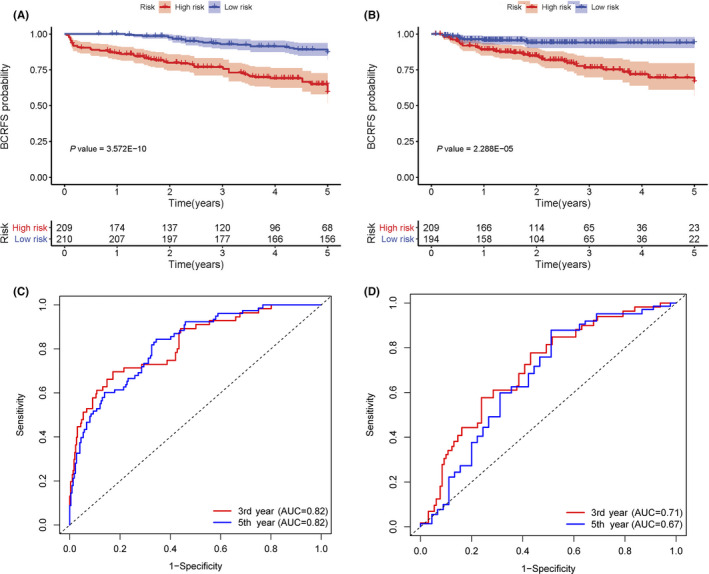
Gene signatures can predict BCRFS in both cohorts. (A) K–M survival curves for the training set indicated that better BCRFS was associated with significantly lower risk score; (B) K–M survival curves for the validation set indicated that better BCRFS was associated with significantly lower risk score; (C) Time‐dependent ROC revealed that the risk score was an excellent predictor for BCRFS in the training set; (D) Time‐dependent ROC revealed that the risk score was an excellent predictor for BCRFS in the validation set. BCRFS, biochemical recurrence‐free survival; K–M, Kaplan–Meier; ROC, receiver operating characteristic

Based on multivariate and univariate Cox regression(CR) models, the risk score, which was adjusted by the clinical variables in both sets, was an independent prognostic factor for BCRFS (*p *< 0.05). Gleason score was prominently associated with BCR in the training set (*n* = 419) (*p *< 0.05) (Table 3). Conversely, no apparent association was observed between BCR and preoperative PSA or cT stage (Table S1; Figure S1). Similarly, in the validation set (*n* = 403), a high Gleason score was notably associated with BCR(*p* < 0.05). Based on Kaplan–Meier survival curves, there were meaningful differences between high‐risk and low‐risk groups for both sets (*p* < 0.001) (Figure 3A,B). According to t‐ROC, the risk score was a strong prognostic factor for BCRFS in the training set (3‐year AUC = 0.82, 5‐year AUC = 0.82) (Figure 3C) and the validation set (3‐year AUC = 0.71, 5‐year AUC = 0.67) (Figure 3D). Python version with scikit‐learn 0.24.0 was used to construct support vector machine (SVM) and a random forest (RF) classifier model to calculate risk score (RF, 3‐year AUC score = 0.73, 5‐year AUC score = 0.76; SVM, 3‐year AUC score = 0.81, 5‐year AUC score = 0.81). The best‐performing model was the CR model, which was selected to calculate the risk score (Table S2).

**TABLE 3:** Univariate and multivariate Cox proportional hazards regression analyses for predicting biochemical recurrence in the training set (*n *= 419) and validation set (*n* = 403)

Variables	Univariate Cox analysis		Multivariate Cox analysis	
	HR (95% CI)	*P* value	HR (95% CI)	*P* value
Training set (*n* = 419)				
Gleason score				
Cont.	1.355 (1.108–1.656)	0.003**	1.426 (1.158–1.757)	<0.001***
Risk score				
Cont.	11.417 (7.160–18.206)	<0.001***	11.584 (7.313–18.349)	<0.001***
Validation set (*n* = 403)				
Gleason score				
Cont.	2.036 (1.540–2.693)	<0.001***	1.639 (1.178–2.281)	0.003**
Risk score				
Cont.	3.884 (2.383–6.331)	<0.001***	2.215 (1.181–4.154)	0.013*

Abbreviations: CI, confidence interval; Cont, continuous; HR, hazard ratio.

* *P* value < 0.05

** *P* value < 0.01

*** *P* value < 0.001.

### 3.4 Establishment of the nomogram

**FIGURE 4:**
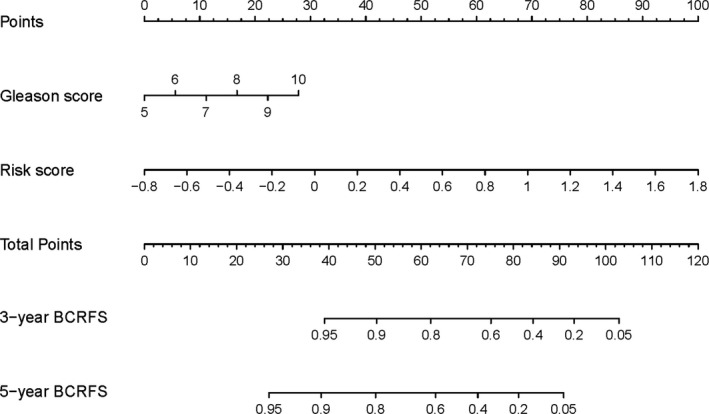
Nomogram prediction of BCRFS probability. The risk score and Gleason score were used to establish the nomogram for predicting 3 and 5‐year BCRFS in the training set. The dominant factor was the risk score. BCRFS, biochemical recurrence‐free survival

**FIGURE 5:**
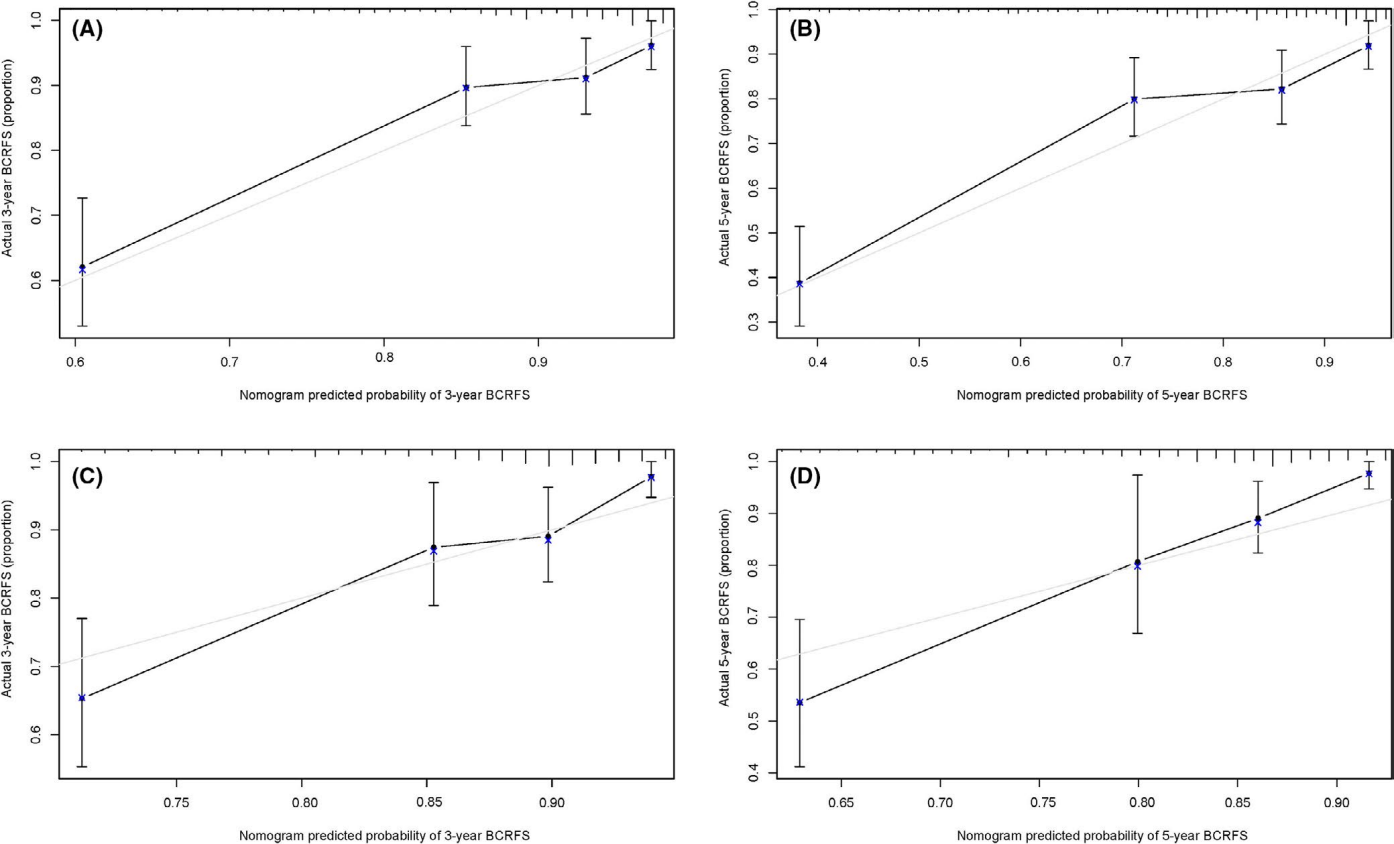
Calibration plots of the nomogram. (A) Three‐year calibration plot of nomogram in the training set; (B) 5‐year calibration plot of nomogram in the training set; (C) 3‐year calibration plot of nomogram in the validation set; (D) 5‐year calibration plot of nomogram in the validation set; the nomogram's performance was excellent for predicting the 3‐year BCRFS and 5‐year BCRFS in both cohorts. BCRFS, biochemical recurrence‐free survival

Following the results of multivariate and univariate Cox analyses, Gleason score and risk score were used to draw a nomogram in the training set. The nomogram predicted the probability of BCRFS in patients with PCa for 3 and 5 years, while the risk score was a dominant factor (Figure 4). The likelihood of BCRFS decreased with an increase in risk score, revealing that our gene signatures might hold promising predictive value for BCRFS. The calibration plots exhibited outstanding conformity between the actual observation and the nomogram prediction for 3‐ and 5‐years BCRFS in the training set (Figure 5A,B) and validation set (Figure 5C,D).

The C‐index of the constructed nomogram for estimating BCRFS was 0.793 in the training set. Compared to the Gleason score (C‐index of 0.588), risk score (C‐index of 0.790), the nomogram showed better predictive accuracy. For the validation set, the constructed nomogram had a C‐index of 0.722 that was also finer to Gleason score (C‐index of 0.676) and risk score (C‐index of 0.710) for BCRFS (Table 4).

**TABLE 4:** The C‐index of the nomogram and other factors in the training and validation sets

Variables	Training set	Validation set
	C‐index	C‐index
Nomogram	0.793	0.722
Gleason score	0.588	0.676
Risk score	0.790	0.710

Abbreviation: C‐index, concordance index.

### 3.5 Bioinformatics analysis

**FIGURE 6:**
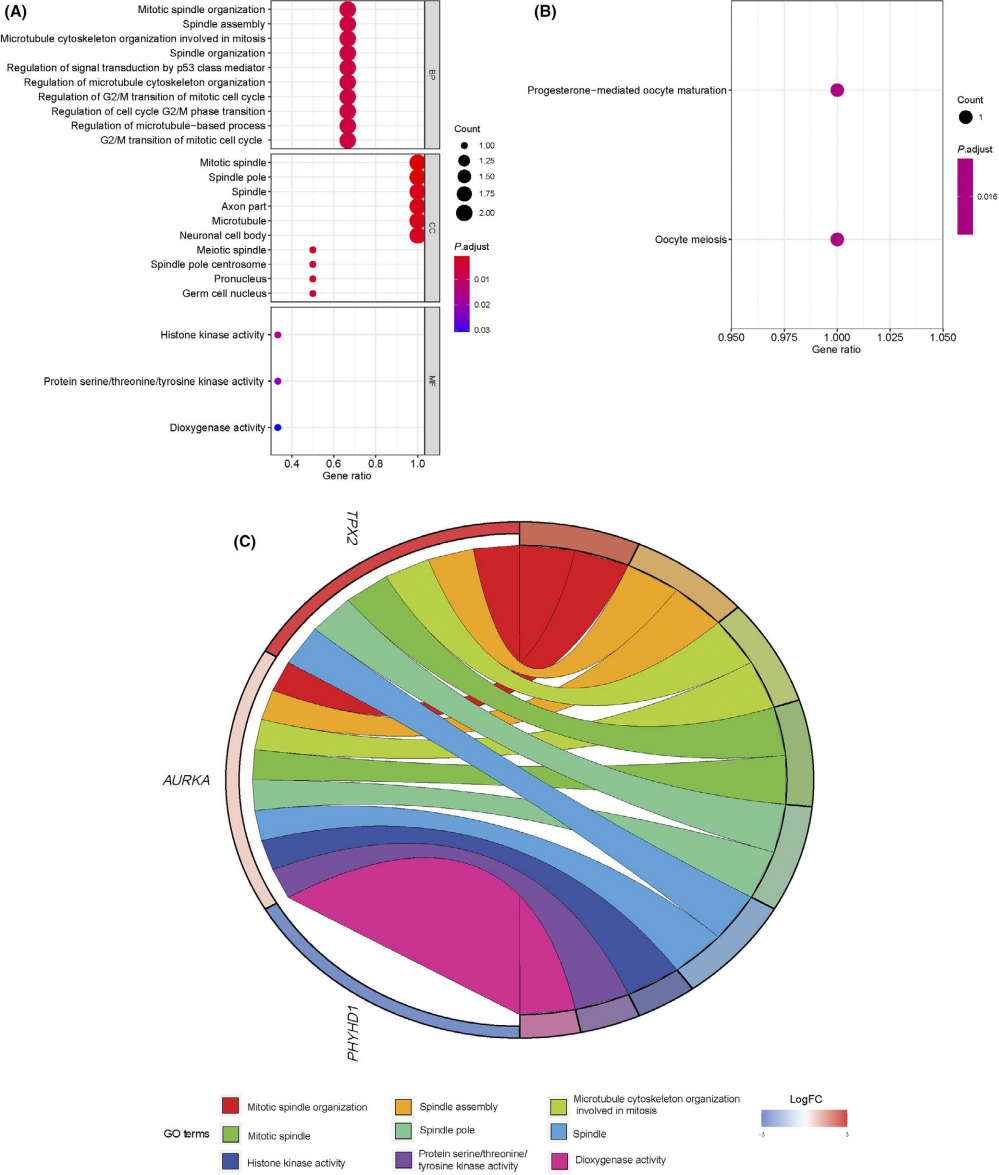
Bioinformatical analysis of three DEGs. (A) Three major categories were included in the bubble plots of GO analysis; (B) two enriched terms of KEGG pathway shown in bubble plot; (C) a chord plot was used to visualize the top three GO terms of BP, CC, and MF, respectively. DEGs, differentially expressed genes; GO, Gene Ontology; BP, biological process; CC, cellular component; MF, molecular function; KEGG, Kyoto Encyclopedia of Gene and Genomes

Above all, three DEGs (PHYHD1, AURKA, and TPX2) between low‐risk (*n* = 210) and high‐risk cases (*n* = 209) were identified using the R package “limma” in the training set, under cut‐off criteria of an adjusted *P* value < 0.05 and |logFC| > 0.5 (Table 5). According to bioinformatics analysis, 145 enriched considerably GO terms belong to the molecular function (MF), biological process (BP), and cellular component (CC) categories (*P* adjusted < 0.05) (Table S3). The most enriched BP terms were associated with mitotic spindle organization, spindle assembly, and microtubule cytoskeleton organization involved in mitosis. The three most dominant terms in CC were mitotic spindle, spindle pole, and spindle. In the MF category, histone kinase activity was the most abundant term, followed by protein serine/threonine/tyrosine kinase activity and dioxygenase activity (Figure 6a). Moreover, two significantly enriched GO terms belong to KEGG categories (*P* adjust < 0.05). As shown in Figure 6B, the notably enriched KEGG pathways of the DEGs were “progesterone‐mediated oocyte maturation” and “Oocyte meiosis.” Ultimate, a chord diagram, was created to measure the relationship between DEGs and GO terms. Figure 6C summarizes the top three pathways enriched in the BP, CC, and MF.

**TABLE 5:** DEGs between low‐risk cases and high‐risk cases in the training set, under cut‐off criteria of |logFC| > 0.5 and adjusted *P* value < 0.05. For each gene, the LogFC, AveExpr, *P* value, and FDR from limma are given

Gene	LogFC	AveExpr	*P* value	FDR
TPX2	4.79279	5.08858	6.63E−55	3.98E−54
PHYHD1	−5.05780	8.83455	3.52E−43	1.06E−42
AURKA	0.79642	3.53217	1.81E−10	3.61E−10

Abbreviations: AveExpr, average expression; DEGs, differentially expressed genes; FDR, false discovery rate adjusted *P* value; LogFC, log fold change.

## 4 DISCUSSION

The current study focuses on appraising the potential prognostic values of gene signatures in BCR using public datasets. Three GEOs associated with BCR are integrated as a training set to obtain optimal gene signatures. Besides, the TCGA dataset serves as an external validation set. The risk score system consisting of 6‐gene signatures is significantly associated with BCRFS by a series of bioinformatical and statistical analyses, which is consistently observed in the validation set. These results indicate that gene signatures have promising predictive value for BCRFS of primary PCa patients after radical therapy. DEGs are explored regarding MF, BP, CC, and KEGG pathways to understand better mechanisms underlying BCR pathogenesis.

Based on uni‐ and multivariate Cox regression models, risk score and Gleason score can predict prognosis in both sets. In contrast, no significant association is observed between BCR and preoperative PSA in the training set. These findings are consistent with previous reports that a high Gleason score was appreciably related to early BCR,whereas factors (i.e., age at diagnosis, preoperative PSA) were not associated with BCRFS.^22^, ^23^ However, our consequences were inconsistent with other reports.^24^, ^25^ This inconsistency may be related to race and radical therapy.

Although radical therapy was a potential prognostic factor for the BCRFS in univariate and subgroup analyses, it was no longer a prognostic factor with multivariate analysis (Figure S1; Table S1). Similar results have been reported in low‐intermediate risk patients with PCa.^26^, ^27^ Nevertheless, this was inconsistent with some previous studies that the therapeutic effect of RP was better than RT, and the probability of BCR after RP was lower than RT.^4^, ^5^ The reason may be that the data came from different datasets and the sample size was small. The experimental results need to be further verified by larger sample size. BCR mainly arises from PCa process itself or as a result of the side effects during treatment. For instance, positive surgical margins (PSM) and lymph node metastases were associated with BCRFS.^28^, ^29^ However, some clinical and pathological parameters (i.e., surgical margin and extracapsular extension) were missing in the training set, so they could not be added for further analysis. In future experiments, more clinical and pathological parameters need to be analyzed. This article focus on the biological characteristics of the disease itself rather than on the therapeutic effect. Furthermore, the predictive contribution of the therapeutic effect was much smaller than the risk score in this article. Thus, the therapeutic effect was not that substantial and did not affect the correctness and reliability of our conclusions.

Several studies highlighted different gene signatures associated with BCR following RP. In a case–control study, a 10‐gene molecular signature(HDDA10) showed superior performance for predicting BCR in PCa patients with RP (AUC = 0.65).^12^ Meanwhile, an original gene signature model predicted 3‐years BCRFS in PCa patients after RP (AUC = 0.836).^11^ In addition, CDO1 promoter methylation was proposed as a feasible predictive biomarker for BCRFS in PCa patients following RP, even though it flunked to reach statistical significance in multivariate analysis.^13^ Our gene signatures may offer a broader range of possibilities for clinical application.

A few biomarkers of our gene signatures have previously been studied in PCa. For example, TPX2, a risk biomarker in our study, positively associated with the BCR of PCa and played an essential role in the proliferation and aggression of PCa.^30^ TPX2 depletion led to the growth inhibition of PCa cells and reduced tumorigenesis.^31^ AURKA, another essential risk biomarker in our study, was correlated with poor prognosis in lethal treatment‐related neuroendocrine prostate cancer.^32^ Also, the inhibition of TPX2 and AURKA stimulated mitotic catastrophe (MC) or apoptosis in PCa cells, and the possible mechanism might be the Glioma pathogenesis‐related protein 1 (GLIPR1) through heat shock cognate protein 70 (Hsc70)‐mediated suppression of TPX2 and AURKA.^33^ Our conclusions show excellent agreement with these results.

Notably, PHYHD1, which has not been studied in PCa, may be involved in the process of BCR. PHYHD1 had been investigated in other tumors. For instance, one research had shown that the DNA methylation level of PHYHD1 was related to the invasion of non‐functioning pituitary adenoma.^34^ However, the underlying mechanism of its action in PCa remains to be established.

There have been several studies that investigated the possible mechanisms of prostate cancer progression. For instance, the centrosome was associated with cell mitosis, and its defects contributed to the change in cellular and gene that accompany the progression, dissemination, and lethality of prostate cancer.^35^ Another study demonstrated that spindle orientation controls cell fate of PCa.^36^ These results resonate well with GO and KEGG results where “mitotic spindle organization” and “Oocyte meiosis” have been the most significantly enriched in prominent GO terms, suggesting their roles as significant progressive pathway signatures in BCR.

This study has the following restrictions. First, this study is restricted by its retrospective proposal and validation. A prospective evaluation would improve the reliability of our findings. Second, experimental evidence to support this conclusion is not yet available and is worthy of further assessment.

In conclusion, the gene signatures in our study have a good fit and discrimination, so does risk score classification, indicating excellent predictive values for BCRFS. Besides, based on risk score and Gleason score, the nomogram can predict 3 and 5‐year BCRFS rates precisely, thus providing evidence of treatment for PCa patients. It is worthy of wider clinical application.

## CONFLICT OF INTEREST

The authors confirm that there are no conflicts of interest.

## ETHICAL APPROVAL STATEMENT

All analyses were based on previously published studies, thus no ethical approval and patient consent are required.

## Supporting information

Fig S1

Table S1

Table S2

Table S3

## Data Availability

The data used to support the findings of this study are available from the GEO (GSE70768, GSE70769, GSE116918, available online: https://www.ncbi.nlm.nih.gov/geo/) and TCGA datasets (available online: https://cancer genome.nih.gov/).
