# Supplementary figures and images for: Gene signatures predict biochemical recurrence‐free survival in primary prostate cancer patients after radical therapy
Source: Cancer Med. 2021 Aug 28;10(18):6492–502. doi: 10.1002/cam4.4092 (PMC8446568; doi:10.1002/cam4.4092)

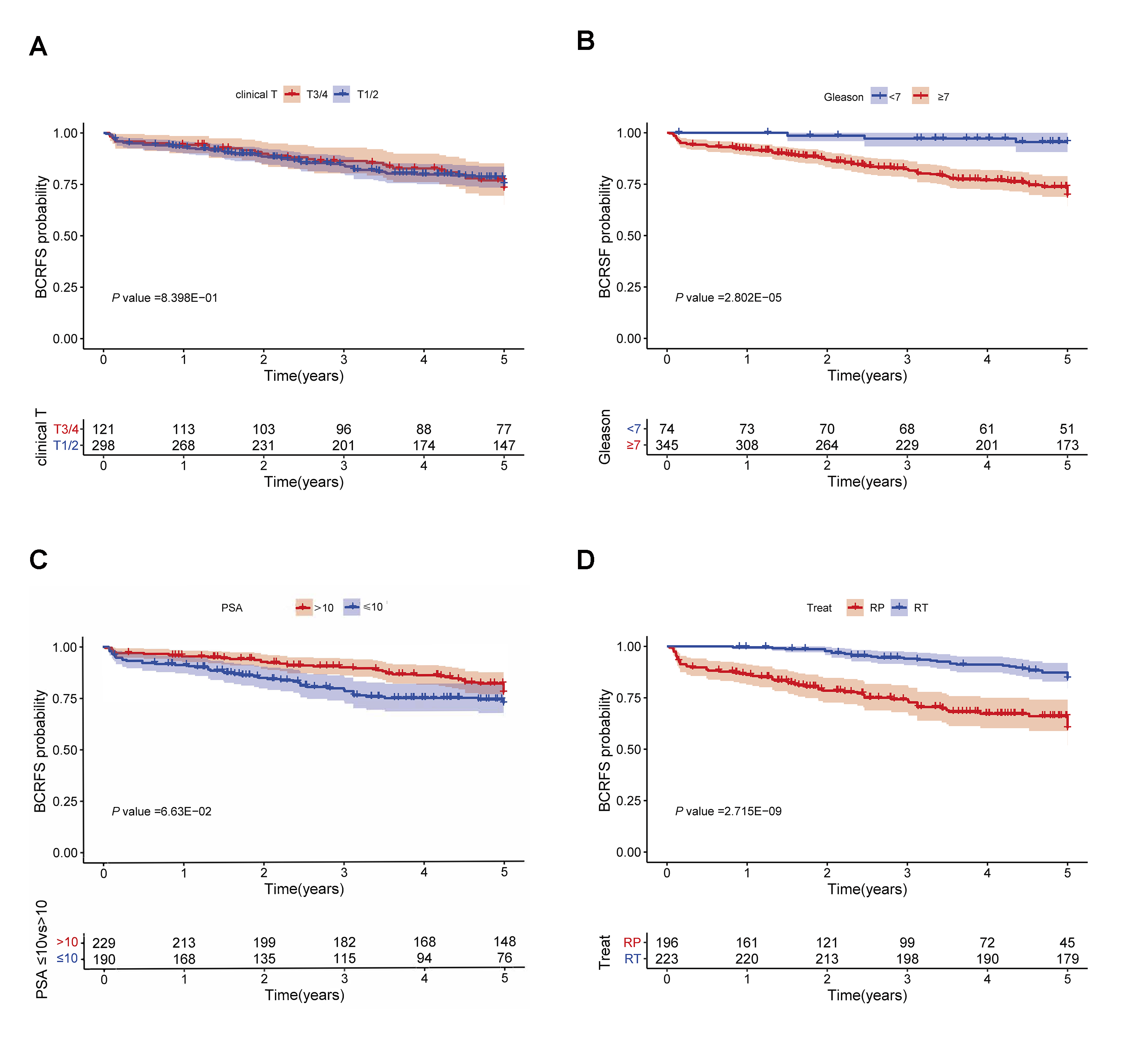

Supplement: Supplementary file 1 — Fig S1 [file CAM4-10-6492-s003.tif]
