# Supplementary material for: Gene signatures predict biochemical recurrence‐free survival in primary prostate cancer patients after radical therapy
Source: Cancer Med. 2021 Aug 28;10(18):6492–502. doi: 10.1002/cam4.4092 (PMC8446568; doi:10.1002/cam4.4092)
Supplement: Supplementary file 2 — Table S1 [file CAM4-10-6492-s001.docx]

| **Table S1. Univariate and multivariate Cox proportional hazards regression analysis for predicting biochemical recurrence in the training set(n=419).** | | | | | |
| --- | --- | --- | --- | --- | --- |
| **Variables** | | **Univariate Cox analysis** | | **Multivariate Cox analysis** | |
|  |  | **HR (95% CI)** | **P value** | **HR (95% CI)** | **P value** |
| Pre-op. PSA | Cont. | 0.995(0.984-1.006) | 0.350 | 0.996(0.984-1.008) | 0.500 |
| cTstage | Cont. | 1.065(0.833−1.361) | 0.614 | 1.207(0.889−1.638) | 0.228 |
| Gleason score | Cont. | 1.355(1.108-1.656) | 0.003** | 1.508(1.154-1.972) | 0.003** |
| Radical therapy | RT vs RP | 0.289(0.188-0.446) | <0.001*** | 0.689(0.315-1.509) | 0.352 |
| Risk score | Cont. | 11.417(7.160-18.206) | <0.001*** | 9.821(5.251-18.367) | <0.001*** |
| Note: Pre-op, preoperative; Cont, continuous; PSA, prostate-specific antigen; cTstage, clinical tumor stage; HR, hazard ratio; CI, confidence interval; RP, radical prostatectomy; vs, versus; RT, radical radiotherapy. **, P value < 0.01; ***, P value < 0.001. | | | | | |
