# Supplementary material for: Gene signatures predict biochemical recurrence‐free survival in primary prostate cancer patients after radical therapy
Source: Cancer Med. 2021 Aug 28;10(18):6492–502. doi: 10.1002/cam4.4092 (PMC8446568; doi:10.1002/cam4.4092)
Supplement: Supplementary file 3 — Table S2 [file CAM4-10-6492-s002.docx]

| **Table S2. AUC of models in predicting BCRSF in the training (n=419) and validation (n=403) sets.** | | | | |
| --- | --- | --- | --- | --- |
| **Model** | **training set** | | **validation set** | |
|  | **3year-AUC** | **5year-AUC** | **3year-AUC** | **5year-AUC** |
| CR | 0.82 | 0.82 | 0.71 | 0.67 |
| SVM | 0.81 | 0.81 | 0.67 | 0.64 |
| RF | 0.73 | 0.76 | 0.58 | 0.46 |
| Note: CR, COX regression; SVM, Support Vector Machine; RF, Random Forest. | | | | |
