# Supplementary material for: Gene signatures predict biochemical recurrence‐free survival in primary prostate cancer patients after radical therapy
Source: Cancer Med. 2021 Aug 28;10(18):6492–502. doi: 10.1002/cam4.4092 (PMC8446568; doi:10.1002/cam4.4092)
Supplement: Supplementary file 4 — Table S3 [file CAM4-10-6492-s004.docx]

**Table S3. 145 enriched considerably GO terms belong to the biological process, cellular component and molecular function categories.**

| **Category** | **GO** | **Description** | **P value** | **P.adjust** | **qvalue** | **Gene** | **Count** |
| --- | --- | --- | --- | --- | --- | --- | --- |
| BP | GO:0007052 | mitotic spindle organization | 9.54E-05 | 0.006554 | 0.000945 | TPX2/AURKA | 2 |
| BP | GO:0051225 | spindle assembly | 9.91E-05 | 0.006554 | 0.000945 | TPX2/AURKA | 2 |
| BP | GO:1902850 | microtubule cytoskeleton organization involved in mitosis | 0.000146 | 0.006554 | 0.000945 | TPX2/AURKA | 2 |
| BP | GO:0007051 | spindle organization | 0.000246 | 0.006554 | 0.000945 | TPX2/AURKA | 2 |
| BP | GO:1901796 | regulation of signal transduction by p53 class mediator | 0.000276 | 0.006554 | 0.000945 | TPX2/AURKA | 2 |
| BP | GO:0070507 | regulation of microtubule cytoskeleton organization | 0.000294 | 0.006554 | 0.000945 | TPX2/AURKA | 2 |
| BP | GO:0010389 | regulation of G2/M transition of mitotic cell cycle | 0.000327 | 0.006554 | 0.000945 | TPX2/AURKA | 2 |
| BP | GO:1902749 | regulation of cell cycle G2/M phase transition | 0.000386 | 0.006554 | 0.000945 | TPX2/AURKA | 2 |
| BP | GO:0032886 | regulation of microtubule-based process | 0.000404 | 0.006554 | 0.000945 | TPX2/AURKA | 2 |
| BP | GO:0000086 | G2/M transition of mitotic cell cycle | 0.000518 | 0.0068 | 0.000981 | TPX2/AURKA | 2 |
| BP | GO:0140014 | mitotic nuclear division | 0.000592 | 0.0068 | 0.000981 | TPX2/AURKA | 2 |
| BP | GO:0044839 | cell cycle G2/M phase transition | 0.000601 | 0.0068 | 0.000981 | TPX2/AURKA | 2 |
| BP | GO:0072331 | signal transduction by p53 class mediator | 0.000606 | 0.0068 | 0.000981 | TPX2/AURKA | 2 |
| BP | GO:0000280 | nuclear division | 0.001402 | 0.013575 | 0.001957 | TPX2/AURKA | 2 |
| BP | GO:0060281 | regulation of oocyte development | 0.001606 | 0.013575 | 0.001957 | AURKA | 1 |
| BP | GO:1905879 | regulation of oogenesis | 0.001606 | 0.013575 | 0.001957 | AURKA | 1 |
| BP | GO:1901990 | regulation of mitotic cell cycle phase transition | 0.001666 | 0.013575 | 0.001957 | TPX2/AURKA | 2 |
| BP | GO:0048285 | organelle fission | 0.001704 | 0.013575 | 0.001957 | TPX2/AURKA | 2 |
| BP | GO:0035404 | histone-serine phosphorylation | 0.001767 | 0.013575 | 0.001957 | AURKA | 1 |
| BP | GO:1901987 | regulation of cell cycle phase transition | 0.001945 | 0.0142 | 0.002048 | TPX2/AURKA | 2 |
| BP | GO:0000212 | meiotic spindle organization | 0.002088 | 0.014514 | 0.002093 | AURKA | 1 |
| BP | GO:0007100 | mitotic centrosome separation | 0.002248 | 0.014919 | 0.002151 | AURKA | 1 |
| BP | GO:0051299 | centrosome separation | 0.002408 | 0.015289 | 0.002205 | AURKA | 1 |
| BP | GO:1903429 | regulation of cell maturation | 0.003691 | 0.022456 | 0.003238 | AURKA | 1 |
| BP | GO:0001556 | oocyte maturation | 0.004172 | 0.024177 | 0.003486 | AURKA | 1 |
| BP | GO:0051642 | centrosome localization | 0.004653 | 0.024177 | 0.003486 | AURKA | 1 |
| BP | GO:0061842 | microtubule organizing center localization | 0.004813 | 0.024177 | 0.003486 | AURKA | 1 |
| BP | GO:0097421 | liver regeneration | 0.004813 | 0.024177 | 0.003486 | AURKA | 1 |
| BP | GO:0007143 | female meiotic nuclear division | 0.004973 | 0.024177 | 0.003486 | AURKA | 1 |
| BP | GO:0071539 | protein localization to centrosome | 0.004973 | 0.024177 | 0.003486 | AURKA | 1 |
| BP | GO:1905508 | protein localization to microtubule organizing center | 0.005133 | 0.024177 | 0.003486 | AURKA | 1 |
| BP | GO:0060236 | regulation of mitotic spindle organization | 0.005934 | 0.026961 | 0.003888 | TPX2 | 1 |
| BP | GO:0016572 | histone phosphorylation | 0.006094 | 0.026961 | 0.003888 | AURKA | 1 |
| BP | GO:0090224 | regulation of spindle organization | 0.006574 | 0.02823 | 0.004071 | TPX2 | 1 |
| BP | GO:0048599 | oocyte development | 0.007374 | 0.028747 | 0.004145 | AURKA | 1 |
| BP | GO:0009948 | anterior/posterior axis specification | 0.008013 | 0.028747 | 0.004145 | AURKA | 1 |
| BP | GO:0072698 | protein localization to microtubule cytoskeleton | 0.008013 | 0.028747 | 0.004145 | AURKA | 1 |
| BP | GO:0009994 | oocyte differentiation | 0.008173 | 0.028747 | 0.004145 | AURKA | 1 |
| BP | GO:0045840 | positive regulation of mitotic nuclear division | 0.008493 | 0.028747 | 0.004145 | AURKA | 1 |
| BP | GO:0044380 | protein localization to cytoskeleton | 0.008812 | 0.028747 | 0.004145 | AURKA | 1 |
| BP | GO:0006977 | DNA damage response, signal transduction by p53 class mediator resulting in cell cycle arrest | 0.008972 | 0.028747 | 0.004145 | AURKA | 1 |
| BP | GO:0090307 | mitotic spindle assembly | 0.008972 | 0.028747 | 0.004145 | TPX2 | 1 |
| BP | GO:0072431 | signal transduction involved in mitotic G1 DNA damage checkpoint | 0.009132 | 0.028747 | 0.004145 | AURKA | 1 |
| BP | GO:1902400 | intracellular signal transduction involved in G1 DNA damage checkpoint | 0.009132 | 0.028747 | 0.004145 | AURKA | 1 |
| BP | GO:0046605 | regulation of centrosome cycle | 0.009291 | 0.028747 | 0.004145 | AURKA | 1 |
| BP | GO:0072413 | signal transduction involved in mitotic cell cycle checkpoint | 0.009451 | 0.028747 | 0.004145 | AURKA | 1 |
| BP | GO:1902402 | signal transduction involved in mitotic DNA damage checkpoint | 0.009451 | 0.028747 | 0.004145 | AURKA | 1 |
| BP | GO:1902403 | signal transduction involved in mitotic DNA integrity checkpoint | 0.009451 | 0.028747 | 0.004145 | AURKA | 1 |
| BP | GO:0031571 | mitotic G1 DNA damage checkpoint | 0.01009 | 0.029341 | 0.004231 | AURKA | 1 |
| BP | GO:0044819 | mitotic G1/S transition checkpoint | 0.01009 | 0.029341 | 0.004231 | AURKA | 1 |
| BP | GO:0044783 | G1 DNA damage checkpoint | 0.010249 | 0.029341 | 0.004231 | AURKA | 1 |
| BP | GO:0051785 | positive regulation of nuclear division | 0.010568 | 0.029673 | 0.004279 | AURKA | 1 |
| BP | GO:2000243 | positive regulation of reproductive process | 0.011047 | 0.030338 | 0.004375 | AURKA | 1 |
| BP | GO:0031100 | animal organ regeneration | 0.011685 | 0.030338 | 0.004375 | AURKA | 1 |
| BP | GO:0072401 | signal transduction involved in DNA integrity checkpoint | 0.011685 | 0.030338 | 0.004375 | AURKA | 1 |
| BP | GO:0072422 | signal transduction involved in DNA damage checkpoint | 0.011685 | 0.030338 | 0.004375 | AURKA | 1 |
| BP | GO:0072395 | signal transduction involved in cell cycle checkpoint | 0.011844 | 0.030338 | 0.004375 | AURKA | 1 |
| BP | GO:0031145 | anaphase-promoting complex-dependent catabolic process | 0.01296 | 0.031923 | 0.004603 | AURKA | 1 |
| BP | GO:0032436 | positive regulation of proteasomal ubiquitin-dependent protein catabolic process | 0.01296 | 0.031923 | 0.004603 | AURKA | 1 |
| BP | GO:0071158 | positive regulation of cell cycle arrest | 0.013119 | 0.031923 | 0.004603 | AURKA | 1 |
| BP | GO:0009798 | axis specification | 0.013597 | 0.032543 | 0.004693 | AURKA | 1 |
| BP | GO:0048477 | oogenesis | 0.013915 | 0.032768 | 0.004725 | AURKA | 1 |
| BP | GO:0032465 | regulation of cytokinesis | 0.014234 | 0.032986 | 0.004756 | AURKA | 1 |
| BP | GO:0010972 | negative regulation of G2/M transition of mitotic cell cycle | 0.01487 | 0.033923 | 0.004892 | AURKA | 1 |
| BP | GO:2000060 | positive regulation of ubiquitin-dependent protein catabolic process | 0.015188 | 0.034115 | 0.004919 | AURKA | 1 |
| BP | GO:0044773 | mitotic DNA damage checkpoint | 0.015506 | 0.034302 | 0.004946 | AURKA | 1 |
| BP | GO:0032091 | negative regulation of protein binding | 0.01646 | 0.034989 | 0.005045 | AURKA | 1 |
| BP | GO:1901800 | positive regulation of proteasomal protein catabolic process | 0.016619 | 0.034989 | 0.005045 | AURKA | 1 |
| BP | GO:1902750 | negative regulation of cell cycle G2/M phase transition | 0.016778 | 0.034989 | 0.005045 | AURKA | 1 |
| BP | GO:0044774 | mitotic DNA integrity checkpoint | 0.016937 | 0.034989 | 0.005045 | AURKA | 1 |
| BP | GO:0030330 | DNA damage response, signal transduction by p53 class mediator | 0.017096 | 0.034989 | 0.005045 | AURKA | 1 |
| BP | GO:0071156 | regulation of cell cycle arrest | 0.017255 | 0.034989 | 0.005045 | AURKA | 1 |
| BP | GO:0007127 | meiosis I | 0.018208 | 0.036415 | 0.005251 | AURKA | 1 |
| BP | GO:0061982 | meiosis I cell cycle process | 0.019001 | 0.037297 | 0.005378 | AURKA | 1 |
| BP | GO:1903052 | positive regulation of proteolysis involved in cellular protein catabolic process | 0.01916 | 0.037297 | 0.005378 | AURKA | 1 |
| BP | GO:0032434 | regulation of proteasomal ubiquitin-dependent protein catabolic process | 0.019477 | 0.037347 | 0.005385 | AURKA | 1 |
| BP | GO:0007098 | centrosome cycle | 0.019794 | 0.037347 | 0.005385 | AURKA | 1 |
| BP | GO:2000134 | negative regulation of G1/S transition of mitotic cell cycle | 0.019953 | 0.037347 | 0.005385 | AURKA | 1 |
| BP | GO:1902807 | negative regulation of cell cycle G1/S phase transition | 0.020904 | 0.038163 | 0.005503 | AURKA | 1 |
| BP | GO:0031023 | microtubule organizing center organization | 0.02122 | 0.038163 | 0.005503 | AURKA | 1 |
| BP | GO:0042770 | signal transduction in response to DNA damage | 0.02122 | 0.038163 | 0.005503 | AURKA | 1 |
| BP | GO:0001889 | liver development | 0.021537 | 0.038163 | 0.005503 | AURKA | 1 |
| BP | GO:0007292 | female gamete generation | 0.021696 | 0.038163 | 0.005503 | AURKA | 1 |
| BP | GO:0061008 | hepaticobiliary system development | 0.022012 | 0.038259 | 0.005517 | AURKA | 1 |
| BP | GO:1903364 | positive regulation of cellular protein catabolic process | 0.022329 | 0.038353 | 0.00553 | AURKA | 1 |
| BP | GO:0000077 | DNA damage checkpoint | 0.02312 | 0.039065 | 0.005633 | AURKA | 1 |
| BP | GO:2000241 | regulation of reproductive process | 0.023278 | 0.039065 | 0.005633 | AURKA | 1 |
| BP | GO:2000058 | regulation of ubiquitin-dependent protein catabolic process | 0.023753 | 0.039408 | 0.005682 | AURKA | 1 |
| BP | GO:0031570 | DNA integrity checkpoint | 0.025017 | 0.040799 | 0.005883 | AURKA | 1 |
| BP | GO:0045931 | positive regulation of mitotic cell cycle | 0.025965 | 0.040799 | 0.005883 | AURKA | 1 |
| BP | GO:0007088 | regulation of mitotic nuclear division | 0.026123 | 0.040799 | 0.005883 | AURKA | 1 |
| BP | GO:0007093 | mitotic cell cycle checkpoint | 0.026281 | 0.040799 | 0.005883 | AURKA | 1 |
| BP | GO:0071695 | anatomical structure maturation | 0.026281 | 0.040799 | 0.005883 | AURKA | 1 |
| BP | GO:0051302 | regulation of cell division | 0.026754 | 0.040799 | 0.005883 | AURKA | 1 |
| BP | GO:1990138 | neuron projection extension | 0.026754 | 0.040799 | 0.005883 | AURKA | 1 |
| BP | GO:0051100 | negative regulation of binding | 0.026912 | 0.040799 | 0.005883 | AURKA | 1 |
| BP | GO:0000910 | cytokinesis | 0.027228 | 0.040799 | 0.005883 | AURKA | 1 |
| BP | GO:0140013 | meiotic nuclear division | 0.027386 | 0.040799 | 0.005883 | AURKA | 1 |
| BP | GO:0048469 | cell maturation | 0.028174 | 0.04155 | 0.005991 | AURKA | 1 |
| BP | GO:0061136 | regulation of proteasomal protein catabolic process | 0.02912 | 0.041985 | 0.006054 | AURKA | 1 |
| BP | GO:2000045 | regulation of G1/S transition of mitotic cell cycle | 0.029277 | 0.041985 | 0.006054 | AURKA | 1 |
| BP | GO:0043902 | positive regulation of multi-organism process | 0.029907 | 0.041985 | 0.006054 | AURKA | 1 |
| BP | GO:0051783 | regulation of nuclear division | 0.029907 | 0.041985 | 0.006054 | AURKA | 1 |
| BP | GO:1903046 | meiotic cell cycle process | 0.029907 | 0.041985 | 0.006054 | AURKA | 1 |
| BP | GO:0031099 | regeneration | 0.031481 | 0.043774 | 0.006312 | AURKA | 1 |
| BP | GO:1902806 | regulation of cell cycle G1/S phase transition | 0.03211 | 0.044227 | 0.006377 | AURKA | 1 |
| BP | GO:0045732 | positive regulation of protein catabolic process | 0.033996 | 0.045747 | 0.006597 | AURKA | 1 |
| BP | GO:1903050 | regulation of proteolysis involved in cellular protein catabolic process | 0.033996 | 0.045747 | 0.006597 | AURKA | 1 |
| BP | GO:0000075 | cell cycle checkpoint | 0.03431 | 0.045747 | 0.006597 | AURKA | 1 |
| BP | GO:0043393 | regulation of protein binding | 0.034467 | 0.045747 | 0.006597 | AURKA | 1 |
| BP | GO:0009952 | anterior/posterior pattern specification | 0.034781 | 0.045748 | 0.006597 | AURKA | 1 |
| BP | GO:0048588 | developmental cell growth | 0.037133 | 0.04774 | 0.006884 | AURKA | 1 |
| BP | GO:0046777 | protein autophosphorylation | 0.03729 | 0.04774 | 0.006884 | AURKA | 1 |
| BP | GO:0060560 | developmental growth involved in morphogenesis | 0.03729 | 0.04774 | 0.006884 | AURKA | 1 |
| BP | GO:0007050 | cell cycle arrest | 0.037603 | 0.04774 | 0.006884 | AURKA | 1 |
| BP | GO:1903362 | regulation of cellular protein catabolic process | 0.039169 | 0.04885 | 0.007044 | AURKA | 1 |
| BP | GO:1901991 | negative regulation of mitotic cell cycle phase transition | 0.039325 | 0.04885 | 0.007044 | AURKA | 1 |
| BP | GO:0051321 | meiotic cell cycle | 0.039482 | 0.04885 | 0.007044 | AURKA | 1 |
| CC | GO:0072686 | mitotic spindle | 3.03E-05 | 0.000727 | NA | TPX2/AURKA | 2 |
| CC | GO:0000922 | spindle pole | 6.88E-05 | 0.000825 | NA | TPX2/AURKA | 2 |
| CC | GO:0005819 | spindle | 0.000309 | 0.002132 | NA | TPX2/AURKA | 2 |
| CC | GO:0033267 | axon part | 0.000374 | 0.002132 | NA | TPX2/AURKA | 2 |
| CC | GO:0005874 | microtubule | 0.000444 | 0.002132 | NA | TPX2/AURKA | 2 |
| CC | GO:0043025 | neuronal cell body | 0.000634 | 0.002537 | NA | TPX2/AURKA | 2 |
| CC | GO:0072687 | meiotic spindle | 0.001116 | 0.003825 | NA | AURKA | 1 |
| CC | GO:0031616 | spindle pole centrosome | 0.001521 | 0.004056 | NA | AURKA | 1 |
| CC | GO:0045120 | pronucleus | 0.001521 | 0.004056 | NA | AURKA | 1 |
| CC | GO:0043073 | germ cell nucleus | 0.001926 | 0.004623 | NA | AURKA | 1 |
| CC | GO:0000780 | condensed nuclear chromosome, centromeric region | 0.002636 | 0.005474 | NA | AURKA | 1 |
| CC | GO:0097431 | mitotic spindle pole | 0.002737 | 0.005474 | NA | AURKA | 1 |
| CC | GO:0051233 | spindle midzone | 0.003446 | 0.006362 | NA | AURKA | 1 |
| CC | GO:0005876 | spindle microtubule | 0.005976 | 0.009561 | NA | AURKA | 1 |
| CC | GO:0045171 | intercellular bridge | 0.005976 | 0.009561 | NA | TPX2 | 1 |
| CC | GO:0000794 | condensed nuclear chromosome | 0.010017 | 0.015026 | NA | AURKA | 1 |
| CC | GO:0000779 | condensed chromosome, centromeric region | 0.011934 | 0.016848 | NA | AURKA | 1 |
| CC | GO:0005814 | centriole | 0.01405 | 0.018734 | NA | AURKA | 1 |
| CC | GO:0005875 | microtubule associated complex | 0.015359 | 0.019401 | NA | AURKA | 1 |
| CC | GO:0030496 | midbody | 0.017472 | 0.020966 | NA | AURKA | 1 |
| CC | GO:0044450 | microtubule organizing center part | 0.018678 | 0.021253 | NA | AURKA | 1 |
| CC | GO:0000775 | chromosome, centromeric region | 0.019482 | 0.021253 | NA | AURKA | 1 |
| CC | GO:0000793 | condensed chromosome | 0.022493 | 0.023471 | NA | AURKA | 1 |
| CC | GO:0098687 | chromosomal region | 0.035088 | 0.035088 | NA | AURKA | 1 |
| MF | GO:0035173 | histone kinase activity | 0.002879 | 0.017275 | 0.006062 | AURKA | 1 |
| MF | GO:0004712 | protein serine/threonine/tyrosine kinase activity | 0.007272 | 0.021816 | 0.007655 | AURKA | 1 |
| MF | GO:0051213 | dioxygenase activity | 0.015348 | 0.030696 | 0.010771 | PHYHD1 | 1 |

Note: BP, biological process; CC, cellular component; MF, molecular function; GO, Gene Ontology.

| **Category** | **ID** | **Description** | ***P* value** | ***P*.adjust** | **qvalue** | **geneID** | **Count** |
| --- | --- | --- | --- | --- | --- | --- | --- |
| BP | GO:0007052 | mitotic spindle organization | 9.54E-05 | 0.006554 | 0.000945 | TPX2/AURKA | 2 |
| BP | GO:0051225 | spindle assembly | 9.91E-05 | 0.006554 | 0.000945 | TPX2/AURKA | 2 |
| BP | GO:1902850 | microtubule cytoskeleton organization involved in mitosis | 0.000146 | 0.006554 | 0.000945 | TPX2/AURKA | 2 |
| BP | GO:0007051 | spindle organization | 0.000246 | 0.006554 | 0.000945 | TPX2/AURKA | 2 |
| BP | GO:1901796 | regulation of signal transduction by p53 class mediator | 0.000276 | 0.006554 | 0.000945 | TPX2/AURKA | 2 |
| BP | GO:0070507 | regulation of microtubule cytoskeleton organization | 0.000294 | 0.006554 | 0.000945 | TPX2/AURKA | 2 |
| BP | GO:0010389 | regulation of G2/M transition of mitotic cell cycle | 0.000327 | 0.006554 | 0.000945 | TPX2/AURKA | 2 |
| BP | GO:1902749 | regulation of cell cycle G2/M phase transition | 0.000386 | 0.006554 | 0.000945 | TPX2/AURKA | 2 |
| BP | GO:0032886 | regulation of microtubule-based process | 0.000404 | 0.006554 | 0.000945 | TPX2/AURKA | 2 |
| BP | GO:0000086 | G2/M transition of mitotic cell cycle | 0.000518 | 0.0068 | 0.000981 | TPX2/AURKA | 2 |
| BP | GO:0140014 | mitotic nuclear division | 0.000592 | 0.0068 | 0.000981 | TPX2/AURKA | 2 |
| BP | GO:0044839 | cell cycle G2/M phase transition | 0.000601 | 0.0068 | 0.000981 | TPX2/AURKA | 2 |
| BP | GO:0072331 | signal transduction by p53 class mediator | 0.000606 | 0.0068 | 0.000981 | TPX2/AURKA | 2 |
| BP | GO:0000280 | nuclear division | 0.001402 | 0.013575 | 0.001957 | TPX2/AURKA | 2 |
| BP | GO:0060281 | regulation of oocyte development | 0.001606 | 0.013575 | 0.001957 | AURKA | 1 |
| BP | GO:1905879 | regulation of oogenesis | 0.001606 | 0.013575 | 0.001957 | AURKA | 1 |
| BP | GO:1901990 | regulation of mitotic cell cycle phase transition | 0.001666 | 0.013575 | 0.001957 | TPX2/AURKA | 2 |
| BP | GO:0048285 | organelle fission | 0.001704 | 0.013575 | 0.001957 | TPX2/AURKA | 2 |
| BP | GO:0035404 | histone-serine phosphorylation | 0.001767 | 0.013575 | 0.001957 | AURKA | 1 |
| BP | GO:1901987 | regulation of cell cycle phase transition | 0.001945 | 0.0142 | 0.002048 | TPX2/AURKA | 2 |
| BP | GO:0000212 | meiotic spindle organization | 0.002088 | 0.014514 | 0.002093 | AURKA | 1 |
| BP | GO:0007100 | mitotic centrosome separation | 0.002248 | 0.014919 | 0.002151 | AURKA | 1 |
| BP | GO:0051299 | centrosome separation | 0.002408 | 0.015289 | 0.002205 | AURKA | 1 |
| BP | GO:1903429 | regulation of cell maturation | 0.003691 | 0.022456 | 0.003238 | AURKA | 1 |
| BP | GO:0001556 | oocyte maturation | 0.004172 | 0.024177 | 0.003486 | AURKA | 1 |
| BP | GO:0051642 | centrosome localization | 0.004653 | 0.024177 | 0.003486 | AURKA | 1 |
| BP | GO:0061842 | microtubule organizing center localization | 0.004813 | 0.024177 | 0.003486 | AURKA | 1 |
| BP | GO:0097421 | liver regeneration | 0.004813 | 0.024177 | 0.003486 | AURKA | 1 |
| BP | GO:0007143 | female meiotic nuclear division | 0.004973 | 0.024177 | 0.003486 | AURKA | 1 |
| BP | GO:0071539 | protein localization to centrosome | 0.004973 | 0.024177 | 0.003486 | AURKA | 1 |
| BP | GO:1905508 | protein localization to microtubule organizing center | 0.005133 | 0.024177 | 0.003486 | AURKA | 1 |
| BP | GO:0060236 | regulation of mitotic spindle organization | 0.005934 | 0.026961 | 0.003888 | TPX2 | 1 |
| BP | GO:0016572 | histone phosphorylation | 0.006094 | 0.026961 | 0.003888 | AURKA | 1 |
| BP | GO:0090224 | regulation of spindle organization | 0.006574 | 0.02823 | 0.004071 | TPX2 | 1 |
| BP | GO:0048599 | oocyte development | 0.007374 | 0.028747 | 0.004145 | AURKA | 1 |
| BP | GO:0009948 | anterior/posterior axis specification | 0.008013 | 0.028747 | 0.004145 | AURKA | 1 |
| BP | GO:0072698 | protein localization to microtubule cytoskeleton | 0.008013 | 0.028747 | 0.004145 | AURKA | 1 |
| BP | GO:0009994 | oocyte differentiation | 0.008173 | 0.028747 | 0.004145 | AURKA | 1 |
| BP | GO:0045840 | positive regulation of mitotic nuclear division | 0.008493 | 0.028747 | 0.004145 | AURKA | 1 |
| BP | GO:0044380 | protein localization to cytoskeleton | 0.008812 | 0.028747 | 0.004145 | AURKA | 1 |
| BP | GO:0006977 | DNA damage response, signal transduction by p53 class mediator resulting in cell cycle arrest | 0.008972 | 0.028747 | 0.004145 | AURKA | 1 |
| BP | GO:0090307 | mitotic spindle assembly | 0.008972 | 0.028747 | 0.004145 | TPX2 | 1 |
| BP | GO:0072431 | signal transduction involved in mitotic G1 DNA damage checkpoint | 0.009132 | 0.028747 | 0.004145 | AURKA | 1 |
| BP | GO:1902400 | intracellular signal transduction involved in G1 DNA damage checkpoint | 0.009132 | 0.028747 | 0.004145 | AURKA | 1 |
| BP | GO:0046605 | regulation of centrosome cycle | 0.009291 | 0.028747 | 0.004145 | AURKA | 1 |
| BP | GO:0072413 | signal transduction involved in mitotic cell cycle checkpoint | 0.009451 | 0.028747 | 0.004145 | AURKA | 1 |
| BP | GO:1902402 | signal transduction involved in mitotic DNA damage checkpoint | 0.009451 | 0.028747 | 0.004145 | AURKA | 1 |
| BP | GO:1902403 | signal transduction involved in mitotic DNA integrity checkpoint | 0.009451 | 0.028747 | 0.004145 | AURKA | 1 |
| BP | GO:0031571 | mitotic G1 DNA damage checkpoint | 0.01009 | 0.029341 | 0.004231 | AURKA | 1 |
| BP | GO:0044819 | mitotic G1/S transition checkpoint | 0.01009 | 0.029341 | 0.004231 | AURKA | 1 |
| BP | GO:0044783 | G1 DNA damage checkpoint | 0.010249 | 0.029341 | 0.004231 | AURKA | 1 |
| BP | GO:0051785 | positive regulation of nuclear division | 0.010568 | 0.029673 | 0.004279 | AURKA | 1 |
| BP | GO:2000243 | positive regulation of reproductive process | 0.011047 | 0.030338 | 0.004375 | AURKA | 1 |
| BP | GO:0031100 | animal organ regeneration | 0.011685 | 0.030338 | 0.004375 | AURKA | 1 |
| BP | GO:0072401 | signal transduction involved in DNA integrity checkpoint | 0.011685 | 0.030338 | 0.004375 | AURKA | 1 |
| BP | GO:0072422 | signal transduction involved in DNA damage checkpoint | 0.011685 | 0.030338 | 0.004375 | AURKA | 1 |
| BP | GO:0072395 | signal transduction involved in cell cycle checkpoint | 0.011844 | 0.030338 | 0.004375 | AURKA | 1 |
| BP | GO:0031145 | anaphase-promoting complex-dependent catabolic process | 0.01296 | 0.031923 | 0.004603 | AURKA | 1 |
| BP | GO:0032436 | positive regulation of proteasomal ubiquitin-dependent protein catabolic process | 0.01296 | 0.031923 | 0.004603 | AURKA | 1 |
| BP | GO:0071158 | positive regulation of cell cycle arrest | 0.013119 | 0.031923 | 0.004603 | AURKA | 1 |
| BP | GO:0009798 | axis specification | 0.013597 | 0.032543 | 0.004693 | AURKA | 1 |
| BP | GO:0048477 | oogenesis | 0.013915 | 0.032768 | 0.004725 | AURKA | 1 |
| BP | GO:0032465 | regulation of cytokinesis | 0.014234 | 0.032986 | 0.004756 | AURKA | 1 |
| BP | GO:0010972 | negative regulation of G2/M transition of mitotic cell cycle | 0.01487 | 0.033923 | 0.004892 | AURKA | 1 |
| BP | GO:2000060 | positive regulation of ubiquitin-dependent protein catabolic process | 0.015188 | 0.034115 | 0.004919 | AURKA | 1 |
| BP | GO:0044773 | mitotic DNA damage checkpoint | 0.015506 | 0.034302 | 0.004946 | AURKA | 1 |
| BP | GO:0032091 | negative regulation of protein binding | 0.01646 | 0.034989 | 0.005045 | AURKA | 1 |
| BP | GO:1901800 | positive regulation of proteasomal protein catabolic process | 0.016619 | 0.034989 | 0.005045 | AURKA | 1 |
| BP | GO:1902750 | negative regulation of cell cycle G2/M phase transition | 0.016778 | 0.034989 | 0.005045 | AURKA | 1 |
| BP | GO:0044774 | mitotic DNA integrity checkpoint | 0.016937 | 0.034989 | 0.005045 | AURKA | 1 |
| BP | GO:0030330 | DNA damage response, signal transduction by p53 class mediator | 0.017096 | 0.034989 | 0.005045 | AURKA | 1 |
| BP | GO:0071156 | regulation of cell cycle arrest | 0.017255 | 0.034989 | 0.005045 | AURKA | 1 |
| BP | GO:0007127 | meiosis I | 0.018208 | 0.036415 | 0.005251 | AURKA | 1 |
| BP | GO:0061982 | meiosis I cell cycle process | 0.019001 | 0.037297 | 0.005378 | AURKA | 1 |
| BP | GO:1903052 | positive regulation of proteolysis involved in cellular protein catabolic process | 0.01916 | 0.037297 | 0.005378 | AURKA | 1 |
| BP | GO:0032434 | regulation of proteasomal ubiquitin-dependent protein catabolic process | 0.019477 | 0.037347 | 0.005385 | AURKA | 1 |
| BP | GO:0007098 | centrosome cycle | 0.019794 | 0.037347 | 0.005385 | AURKA | 1 |
| BP | GO:2000134 | negative regulation of G1/S transition of mitotic cell cycle | 0.019953 | 0.037347 | 0.005385 | AURKA | 1 |
| BP | GO:1902807 | negative regulation of cell cycle G1/S phase transition | 0.020904 | 0.038163 | 0.005503 | AURKA | 1 |
| BP | GO:0031023 | microtubule organizing center organization | 0.02122 | 0.038163 | 0.005503 | AURKA | 1 |
| BP | GO:0042770 | signal transduction in response to DNA damage | 0.02122 | 0.038163 | 0.005503 | AURKA | 1 |
| BP | GO:0001889 | liver development | 0.021537 | 0.038163 | 0.005503 | AURKA | 1 |
| BP | GO:0007292 | female gamete generation | 0.021696 | 0.038163 | 0.005503 | AURKA | 1 |
| BP | GO:0061008 | hepaticobiliary system development | 0.022012 | 0.038259 | 0.005517 | AURKA | 1 |
| BP | GO:1903364 | positive regulation of cellular protein catabolic process | 0.022329 | 0.038353 | 0.00553 | AURKA | 1 |
| BP | GO:0000077 | DNA damage checkpoint | 0.02312 | 0.039065 | 0.005633 | AURKA | 1 |
| BP | GO:2000241 | regulation of reproductive process | 0.023278 | 0.039065 | 0.005633 | AURKA | 1 |
| BP | GO:2000058 | regulation of ubiquitin-dependent protein catabolic process | 0.023753 | 0.039408 | 0.005682 | AURKA | 1 |
| BP | GO:0031570 | DNA integrity checkpoint | 0.025017 | 0.040799 | 0.005883 | AURKA | 1 |
| BP | GO:0045931 | positive regulation of mitotic cell cycle | 0.025965 | 0.040799 | 0.005883 | AURKA | 1 |
| BP | GO:0007088 | regulation of mitotic nuclear division | 0.026123 | 0.040799 | 0.005883 | AURKA | 1 |
| BP | GO:0007093 | mitotic cell cycle checkpoint | 0.026281 | 0.040799 | 0.005883 | AURKA | 1 |
| BP | GO:0071695 | anatomical structure maturation | 0.026281 | 0.040799 | 0.005883 | AURKA | 1 |
| BP | GO:0051302 | regulation of cell division | 0.026754 | 0.040799 | 0.005883 | AURKA | 1 |
| BP | GO:1990138 | neuron projection extension | 0.026754 | 0.040799 | 0.005883 | AURKA | 1 |
| BP | GO:0051100 | negative regulation of binding | 0.026912 | 0.040799 | 0.005883 | AURKA | 1 |
| BP | GO:0000910 | cytokinesis | 0.027228 | 0.040799 | 0.005883 | AURKA | 1 |
| BP | GO:0140013 | meiotic nuclear division | 0.027386 | 0.040799 | 0.005883 | AURKA | 1 |
| BP | GO:0048469 | cell maturation | 0.028174 | 0.04155 | 0.005991 | AURKA | 1 |
| BP | GO:0061136 | regulation of proteasomal protein catabolic process | 0.02912 | 0.041985 | 0.006054 | AURKA | 1 |
| BP | GO:2000045 | regulation of G1/S transition of mitotic cell cycle | 0.029277 | 0.041985 | 0.006054 | AURKA | 1 |
| BP | GO:0043902 | positive regulation of multi-organism process | 0.029907 | 0.041985 | 0.006054 | AURKA | 1 |
| BP | GO:0051783 | regulation of nuclear division | 0.029907 | 0.041985 | 0.006054 | AURKA | 1 |
| BP | GO:1903046 | meiotic cell cycle process | 0.029907 | 0.041985 | 0.006054 | AURKA | 1 |
| BP | GO:0031099 | regeneration | 0.031481 | 0.043774 | 0.006312 | AURKA | 1 |
| BP | GO:1902806 | regulation of cell cycle G1/S phase transition | 0.03211 | 0.044227 | 0.006377 | AURKA | 1 |
| BP | GO:0045732 | positive regulation of protein catabolic process | 0.033996 | 0.045747 | 0.006597 | AURKA | 1 |
| BP | GO:1903050 | regulation of proteolysis involved in cellular protein catabolic process | 0.033996 | 0.045747 | 0.006597 | AURKA | 1 |
| BP | GO:0000075 | cell cycle checkpoint | 0.03431 | 0.045747 | 0.006597 | AURKA | 1 |
| BP | GO:0043393 | regulation of protein binding | 0.034467 | 0.045747 | 0.006597 | AURKA | 1 |
| BP | GO:0009952 | anterior/posterior pattern specification | 0.034781 | 0.045748 | 0.006597 | AURKA | 1 |
| BP | GO:0048588 | developmental cell growth | 0.037133 | 0.04774 | 0.006884 | AURKA | 1 |
| BP | GO:0046777 | protein autophosphorylation | 0.03729 | 0.04774 | 0.006884 | AURKA | 1 |
| BP | GO:0060560 | developmental growth involved in morphogenesis | 0.03729 | 0.04774 | 0.006884 | AURKA | 1 |
| BP | GO:0007050 | cell cycle arrest | 0.037603 | 0.04774 | 0.006884 | AURKA | 1 |
| BP | GO:1903362 | regulation of cellular protein catabolic process | 0.039169 | 0.04885 | 0.007044 | AURKA | 1 |
| BP | GO:1901991 | negative regulation of mitotic cell cycle phase transition | 0.039325 | 0.04885 | 0.007044 | AURKA | 1 |
| BP | GO:0051321 | meiotic cell cycle | 0.039482 | 0.04885 | 0.007044 | AURKA | 1 |
| CC | GO:0072686 | mitotic spindle | 3.03E-05 | 0.000727 | NA | TPX2/AURKA | 2 |
| CC | GO:0000922 | spindle pole | 6.88E-05 | 0.000825 | NA | TPX2/AURKA | 2 |
| CC | GO:0005819 | spindle | 0.000309 | 0.002132 | NA | TPX2/AURKA | 2 |
| CC | GO:0033267 | axon part | 0.000374 | 0.002132 | NA | TPX2/AURKA | 2 |
| CC | GO:0005874 | microtubule | 0.000444 | 0.002132 | NA | TPX2/AURKA | 2 |
| CC | GO:0043025 | neuronal cell body | 0.000634 | 0.002537 | NA | TPX2/AURKA | 2 |
| CC | GO:0072687 | meiotic spindle | 0.001116 | 0.003825 | NA | AURKA | 1 |
| CC | GO:0031616 | spindle pole centrosome | 0.001521 | 0.004056 | NA | AURKA | 1 |
| CC | GO:0045120 | pronucleus | 0.001521 | 0.004056 | NA | AURKA | 1 |
| CC | GO:0043073 | germ cell nucleus | 0.001926 | 0.004623 | NA | AURKA | 1 |
| CC | GO:0000780 | condensed nuclear chromosome, centromeric region | 0.002636 | 0.005474 | NA | AURKA | 1 |
| CC | GO:0097431 | mitotic spindle pole | 0.002737 | 0.005474 | NA | AURKA | 1 |
| CC | GO:0051233 | spindle midzone | 0.003446 | 0.006362 | NA | AURKA | 1 |
| CC | GO:0005876 | spindle microtubule | 0.005976 | 0.009561 | NA | AURKA | 1 |
| CC | GO:0045171 | intercellular bridge | 0.005976 | 0.009561 | NA | TPX2 | 1 |
| CC | GO:0000794 | condensed nuclear chromosome | 0.010017 | 0.015026 | NA | AURKA | 1 |
| CC | GO:0000779 | condensed chromosome, centromeric region | 0.011934 | 0.016848 | NA | AURKA | 1 |
| CC | GO:0005814 | centriole | 0.01405 | 0.018734 | NA | AURKA | 1 |
| CC | GO:0005875 | microtubule associated complex | 0.015359 | 0.019401 | NA | AURKA | 1 |
| CC | GO:0030496 | midbody | 0.017472 | 0.020966 | NA | AURKA | 1 |
| CC | GO:0044450 | microtubule organizing center part | 0.018678 | 0.021253 | NA | AURKA | 1 |
| CC | GO:0000775 | chromosome, centromeric region | 0.019482 | 0.021253 | NA | AURKA | 1 |
| CC | GO:0000793 | condensed chromosome | 0.022493 | 0.023471 | NA | AURKA | 1 |
| CC | GO:0098687 | chromosomal region | 0.035088 | 0.035088 | NA | AURKA | 1 |
| MF | GO:0035173 | histone kinase activity | 0.002879 | 0.017275 | 0.006062 | AURKA | 1 |
| MF | GO:0004712 | protein serine/threonine/tyrosine kinase activity | 0.007272 | 0.021816 | 0.007655 | AURKA | 1 |
| MF | GO:0051213 | dioxygenase activity | 0.015348 | 0.030696 | 0.010771 | PHYHD1 | 1 |

Note: BP, biological process; CC, cellular component; MF, molecular function; GO, Gene Ontology.
